# Microbial Identification Using rRNA Operon Region: Database and Tool for Metataxonomics with Long-Read Sequence

**DOI:** 10.1128/spectrum.02017-21

**Published:** 2022-03-30

**Authors:** Donghyeok Seol, Jin Soo Lim, Samsun Sung, Young Ho Lee, Misun Jeong, Seoae Cho, Woori Kwak, Heebal Kim

**Affiliations:** a eGnome, Inc, Seoul, Republic of Korea; b Department of Agricultural Biotechnology and Research Institute of Agriculture and Life Sciences, Seoul National University, Seoul, Republic of Korea; c Interdisciplinary Program in Bioinformatics, Seoul National University, Seoul, Republic of Korea; d Hoonygen, Seoul, Republic of Korea; e Gencube Plus, Seoul, Republic of Korea; Nanchang University

**Keywords:** Oxford Nanopore Technologies, Pacific Biosciences, curated database, full-length 16S rRNA, metagenomics, microbiome, *rrn* operon

## Abstract

Recent development of long-read sequencing platforms has enabled researchers to explore bacterial community structure through analysis of full-length 16S rRNA gene (∼1,500 bp) or 16S-ITS-23S rRNA operon region (∼4,300 bp), resulting in higher taxonomic resolution than short-read sequencing platforms. Despite the potential of long-read sequencing in metagenomics, resources and protocols for this technology are scarce. Here, we describe MIrROR, the database and analysis tool for metataxonomics using the bacterial 16S-ITS-23S rRNA operon region. We collected 16S-ITS-23S rRNA operon sequences extracted from bacterial genomes from NCBI GenBank and performed curation. A total of 97,781 16S-ITS-23S rRNA operon sequences covering 9,485 species from 43,653 genomes were obtained. For user convenience, we provide an analysis tool based on a mapping strategy that can be used for taxonomic profiling with MIrROR database. To benchmark MIrROR, we compared performance against publicly available databases and tool with mock communities and simulated data sets. Our platform showed promising results in terms of the number of species covered and the accuracy of classification. To encourage active 16S-ITS-23S rRNA operon analysis in the field, BLAST function and taxonomic profiling results with 16S-ITS-23S rRNA operon studies, which have been reported as BioProject on NCBI are provided. MIrROR (http://mirror.egnome.co.kr/) will be a useful platform for researchers who want to perform high-resolution metagenome analysis with a cost-effective sequencer such as MinION from Oxford Nanopore Technologies.

**IMPORTANCE** Metabarcoding is a powerful tool to investigate community diversity in an economic and efficient way by amplifying a specific gene marker region. With the advancement of long-read sequencing technologies, the field of metabarcoding has entered a new phase. The technologies have brought a need for development in several areas, including new markers that long-read can cover, database for the markers, tools that reflect long-read characteristics, and compatibility with downstream analysis tools. By constructing MIrROR, we met the need for a database and tools for the 16S-ITS-23S rRNA operon region, which has recently been shown to have sufficient resolution at the species level. Bacterial community analysis using the 16S-ITS-23S rRNA operon region with MIrROR will provide new insights from various research fields.

## INTRODUCTION

The 16S rRNA gene is widely used as the gold standard marker for bacterial community profiling (1–4). Along with its ubiquity, the rare occurrence of horizontal gene transfer (5), sufficient taxonomic information from hypervariable regions (V1 to V9), and a universally conserved region that can be used as a PCR binding site have made this gene appropriate as a taxonomic marker. With features in this gene alone, researchers have been able to efficiently and inexpensively obtain taxonomic profiles without having to analyze every genomic region derived from shotgun sequencing. However, analysis of the full-length 16S rRNA gene (∼1,500 bp) cannot be performed with short-read sequencing platforms such as Illumina’s MiSeq, which are limited to reads of up to several hundred bases. Therefore, only partial sequences (V1 and V2 [6], V3 and V4 [7], V4 [4], and V4 and V5 [8]), which allow maximum use of the informative site under the length restriction, are targeted for community analysis. Such a loss of information has resulted in ambiguous profiling that cannot even guarantee genus-level resolutions at times (9). Moreover, bacterial communities vary greatly depending on which variable region is targeted, and there were cases in which a specific taxon was missing due to variants in the primer binding site or the absence of sequences to be compared in the database (10–13). For this reason, additional analysis was required to find candidate causative species within differentially abundant genera in profiling results (14).

Advances in long-read sequencing are expanding our knowledge in various fields regardless of genomics or transcriptomics, such as high-quality genome assembly, transcript isoform identification, and detection of base modification without other processing (15). Likewise, in metagenomics, short-read sequencing-based metabarcoding using only part of the 16S rRNA gene has changed to long-read sequencing, which can target the full-length 16S rRNA gene, improving taxonomic resolution up to species or strain (16). However, the sequencing platform capable of producing much longer reads leaves room for improvement in comparing 1.5-kb sequences for species classification.

Recently, there has been an attempt to amplify the 16S-ITS-23S rRNA operon sequence (∼4,300 bp), and this long fragment with more informative sites enables more accurate analysis at the species level (17, 18). Since then, studies in which rRNA operon is appropriate as the taxonomic marker for bacteria have been emerging (19, 20). However, curated databases and suitable analysis pipelines for rRNA operon analysis are still not available (18, 21). Existing rRNA sequence databases such as SILVA (22), Greengenes (23), and RDP (24) have only single-split rRNA information without linking internal transcribed spacer (ITS) region, so they can cover only up to full-length 16S rRNA analysis.

Here, we present MIrROR, which consists of a rRNA operon database curated through several quality control (QC) steps and analysis tool that profiles bacterial communities based on mapping strategy. To cover as many species as possible, all bacterial genomes available in the National Center for Biotechnology Information (NCBI) were collected (25), and quality control was performed on taxonomy misassignment or contaminants, which are problematic in the public repository (26). For the mapping-based tool, we considered the length of the alignment block and the number of mismatches and configured the output to be used for subsequent analysis. Finally, we evaluated the performance of MIrROR by simulating Nanopore reads from four major human body sites (gut, skin, vagina, and oral cavity) and sequencing two mock communities. We then compared it with the publicly available databases rrn_DBv2 (27), 16S-23S-rRNA encoding region database (16_mar) (28), SILVA (22), and Greengenes (23) and with the other metagenomic classification tool, Kraken2 (29).

## RESULTS

**FIG 1:**
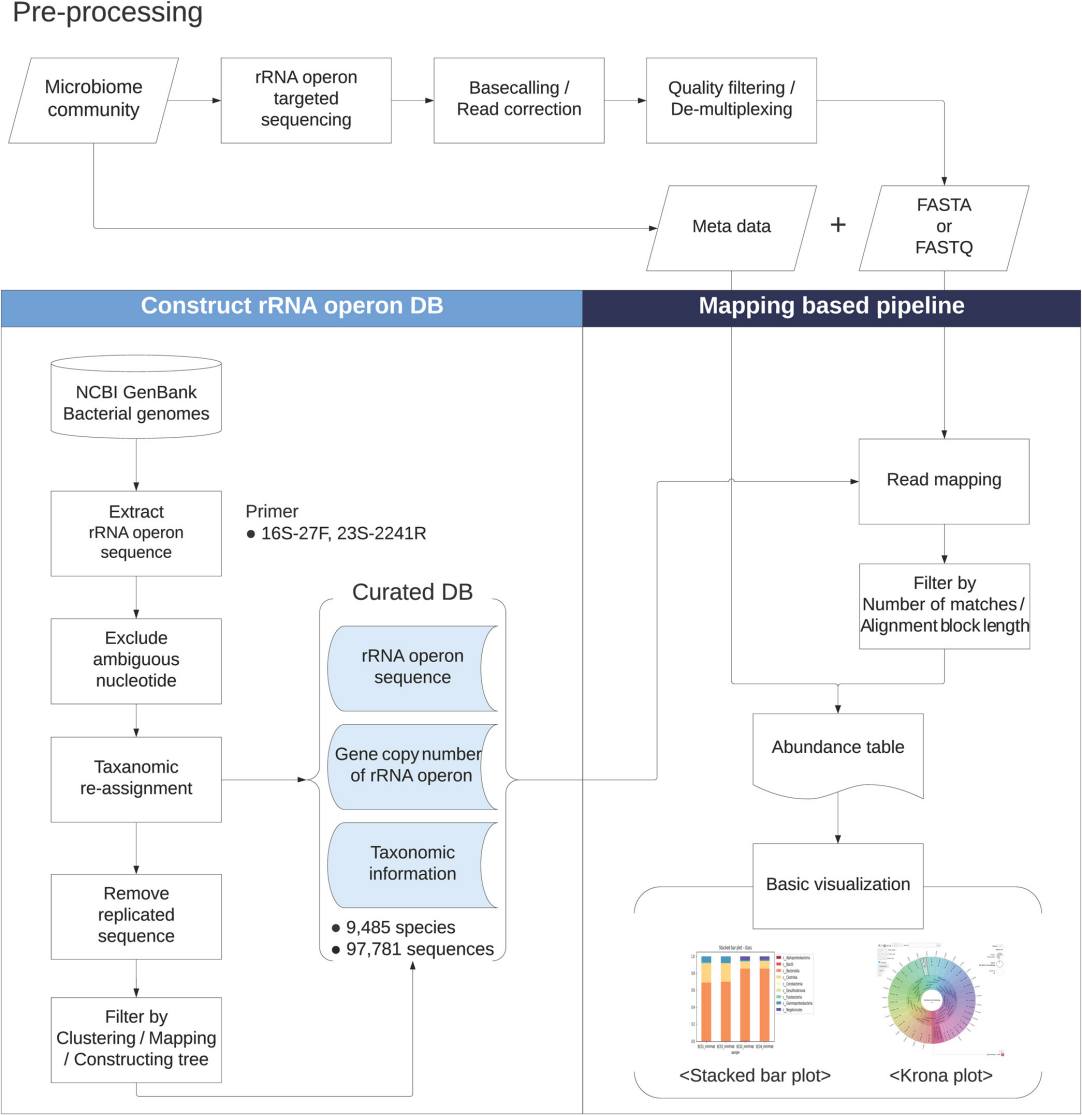
Schematic workflow for constructing MIrROR. Squares, parallelograms, cylinder, and rectangle with a curved bottom shape represent “Process,” “Data,” “Database,” and “Report,” respectively. Shapes shaded in blue represent MIrROR database (DB). The flowchart was generated by Lucidchart (https://www.lucidchart.com/).

MIrROR consists of the 16S-ITS-23S rRNA operon database and the analysis tool for 16S-ITS-23S rRNA operon sequences. The curation process for database construction and the pipeline of the tool are summarized in Fig. 1. The software and databases used during the process are listed in Table 1.

**TABLE 1:** Software and databases used for constructing MIrROR

Step	Software/database	Utility	Version	Reference
Database	NCBI GenBank	Get genomes	8 January 2020	44
	EMBOSS-primersearch	Extract 16S-ITS-23S rRNA operon sequence	6.6.0.0	45
	GTDB	Taxonomic reassignment	r89	73
	GTDB-Tk	Taxonomic reassignment	1.2.0	46
	Prodigal	Dependency of GTDB-Tk	2.6.3	74
	HMMER	Dependency of GTDB-Tk	3.1b2	75
	pplacer	Dependency of GTDB-Tk	1.1.alpha19	76
	FastANI	Dependency of GTDB-Tk	1.3	77
	Cd-hit-est	Sequence clustering	4.8.1	47
	Clustal Omega	Multiple sequence alignment	1.2.4	49
	IQ-tree	Constructing tree	2.0.6	50
	BLAST	Quality control filtering	2.10.1	51
Project	Entrez Direct	Get BioProject	13.9	52
Analysis tool	Minimap2	Read mapping	2.17	53
	KronaTools	Visualization	2.7.1	54
	OTUsamples2krona	Visualization	0.2.2	^*a*^
	Pandas (Python package)	Visualization	0.24.2	^*b*^
	Matplotlib (Python package)	Visualization	2.1.2	^*c*^

a https://github.com/GenomicaMicrob/OTUsamples2krona.

b https://pandas.pydata.org/.

c https://matplotlib.org/.

### MIrROR database.

We used a total of 459,136 genomes from NCBI GenBank for the construction of MIrROR database (accessed 8 January 2020). When extracting 16S-ITS-23S rRNA operon sequences from the genome, we did not use the rRNA sequence prediction tool but identified regions amplified by the universal 16S-ITS-23S primer set for the following reasons: (i) The target region of the most commonly used primer set does not cover the whole 23S rRNA gene sequence. Therefore, it is necessary to check whether there is a primer binding site regardless of the completeness of the 23S rRNA sequence. Conversely, even if the 23S rRNA sequence is complete, it may not be amplified when primer binding sites have variants. (ii) Despite the specificity of the universal primer, sequences other than the 16S-ITS-23S rRNA operon region near 4,300 bp in bacterial genomes may be amplified. Therefore, it is necessary to check whether unexpected amplicons that can affect community analysis are included. Only genomes without ambiguous nucleotides in 16S-ITS-23S rRNA operon sequences were considered for the next step. As a result of whole genome-based taxonomic reassignment, 123,317 genomes were assigned at the species level. Within species, 20,285 redundant sequences from 99,960 genomes were removed. Among the remaining genomes, 3,365 genomes containing 16S-ITS-23S rRNA operon sequences similar to those of other species were removed.

**FIG 2:**
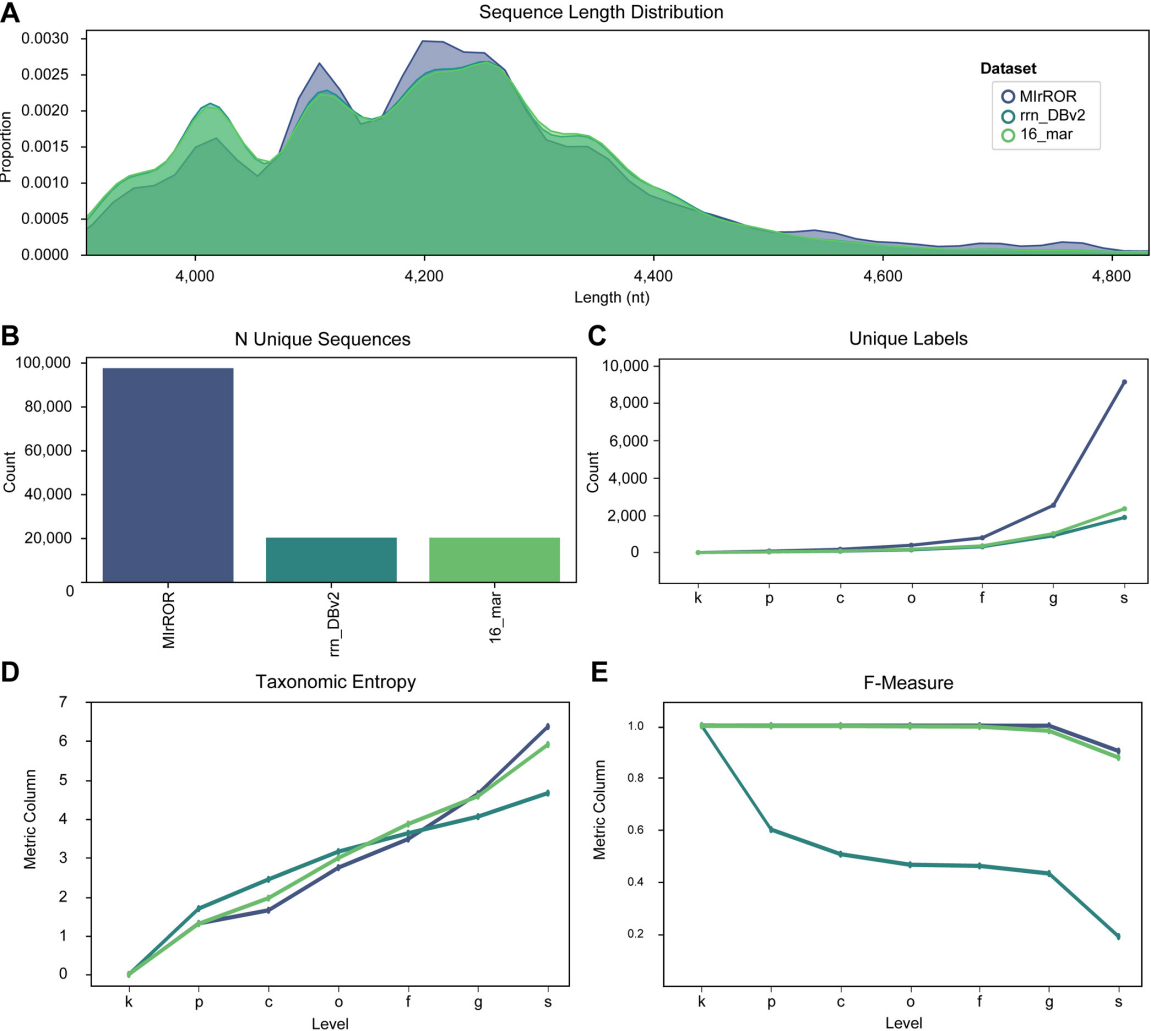
Summary of basic statistics for three 16S-ITS-23S rRNA operon databases. (A) Sequence length distributions. (B) Number of unique sequences. (C) Number of unique taxonomic labels. (D) Taxonomic entropy, which is the measurement of both richness and evenness for taxonomic information. (E) F-measure calculated on naive Bayes-based classification. The taxonomic rank labels on *x* axis are as follows: k, kingdom; p, phylum; c, class; o, order; f, family; g, genus; s, species.

After several curation steps, we finally obtained 97,781 rRNA operon sequences covering 9,485 species from 43,653 genomes as MIrROR database (release 01). The sequence length distribution of MIrROR is not significantly different from the previously reported databases rrn_DBv2 and 16_mar (Fig. 2A). MIrROR, however, has four times more sequences and three times more species labels than other databases (Fig. 2B and C), as well as the highest species-level taxonomic entropy (Fig. 2D). In QIIME2’s fit-classifier-based classification performance test, MIrROR and 16_mar were found to have similar accuracies (Fig. 2E).

### Web interface.

**FIG 3:**
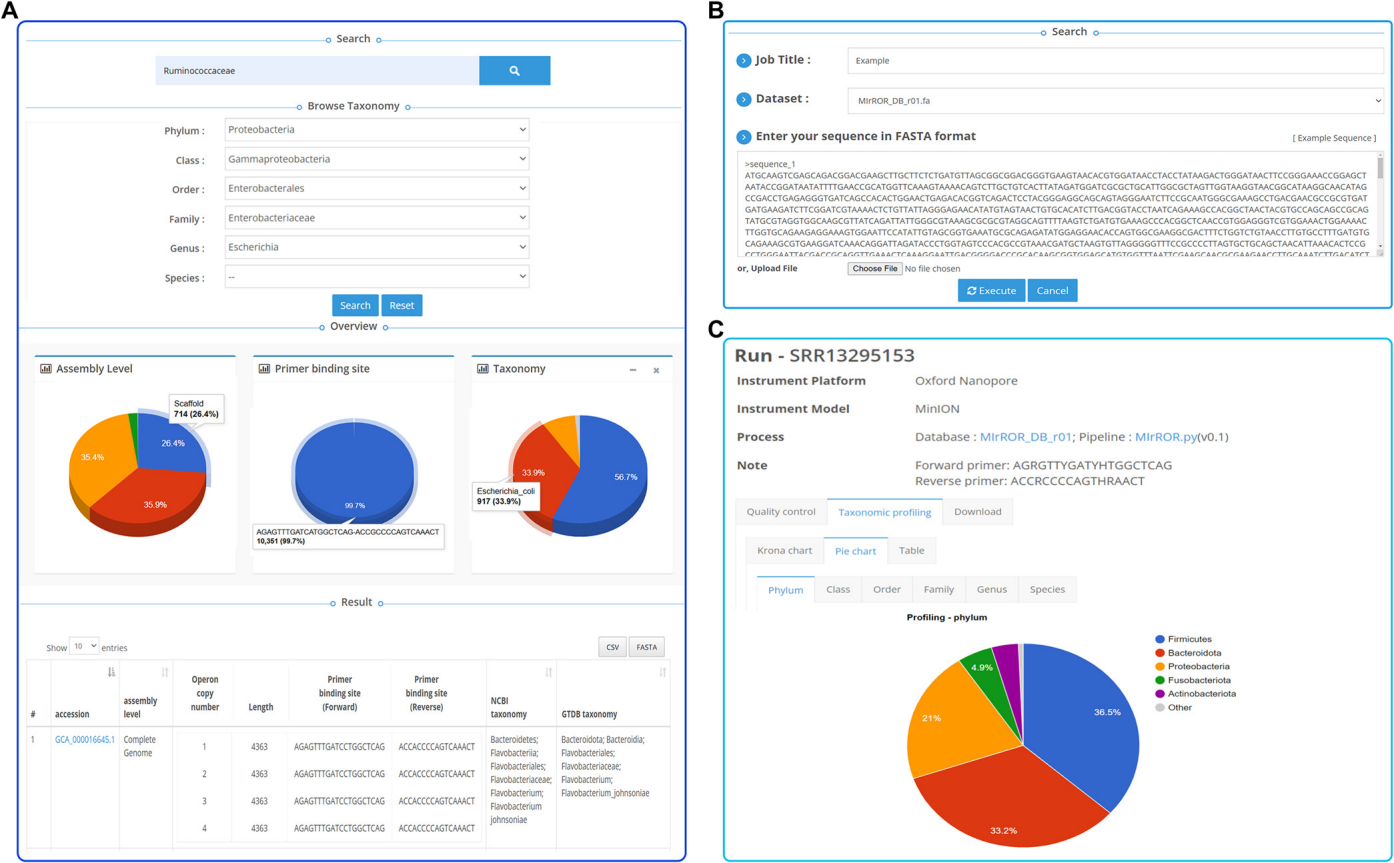
Data browsing and BLAST search function in the MIrROR website. (A) Browsing interface in MIrROR. When users search through NCBI accession number or taxonomic name, an overview consisting of “Assembly Level,” “Primer binding site,” and “Taxonomy” and information on the 16S-ITS-23S rRNA operon by a genome are displayed. (B) BLAST search function in MIrROR. Users can align their 16S-ITS-23S rRNA operon sequences against MIrROR to find the closest matches. (C) Screenshot showing profiling results at the phylum level based on MIrROR (http://mirror.egnome.co.kr/project/133/run/).

Through the website (http://mirror.egnome.co.kr/), MIrROR provides information on the 16S-ITS-23S rRNA sequences that passed the curation for each taxon. Users can browse specific taxa in two ways. First, the database can be searched through NCBI assembly accession or taxonomic name. Taxonomic name searches support both NCBI and GTDB taxonomic information. Second, if users do not know the taxonomic name, they can browse taxa hierarchically. Search results provide gene sequences and information such as the length of 16S-ITS-23S rRNA operon and variant within the primer binding site for each genome. Users can download metadata and 16S-ITS-23S rRNA operon sequences about the taxa of interest in CSV and FASTA files, respectively (Fig. 3A).

To quickly provide users with taxonomic information for several 16S-ITS-23S sequences, MIrROR incorporates a BLAST search function. Users can use BLAST by inputting sequences in FASTA format. They are aligned against the MIrROR database, and the results of GTDB-based taxonomic profiling are returned in the order of the bit score (Fig. 3B).

MIrROR provides taxonomic profiling for all reported metataxonomic samples targeting 16S-ITS-23S rRNA operon sequences. It is organized hierarchically in the following order: BioProject, BioSample, SRA Experiment, and SRA Runs. Users can explore the metadata for each section. In the last section, SRA Runs, the table with QC filtering and relative abundance results is presented (Fig. 3C).

### Assessing taxonomic analysis through sequencing data of mock community.

We tested the performance of MIrROR on two mock communities (ZymoBIOMICS): MOCK1 containing 8 bacterial species and MOCK2 containing 14 bacterial species (Table 2). The taxa were reassigned from Bacillus subtilis, Escherichia coli, and Fusobacterium nucleatum to Bacillus marinus, Escherichia flexneri, and Fusobacterium animalis, respectively.

**TABLE 2:** Description of bacterial mock communities^*a*^

Mock community	Product name	Catalog no.	Species	Species (GTDB)	Theoretical composition (16S-23S rRNA operon) (%)	NCBI accession	Reference	
MOCK1	ZymoBIOMICS Microbial Community Standard	D6300	Bacillus subtilis	Bacillus marinus	17.4	SRR8029985, SRR8029986		18
			Enterococcus faecalis		9.9			
			Escherichia coli	Escherichia flexneri	10.1			
			Lactobacillus fermentum		18.4			
			Listeria monocytogenes		14.1			
			Pseudomonas aeruginosa		4.2			
			Salmonella enterica		10.4			
			Staphylococcus aureus		15.5			
			Cryptococcus neoformans	NA^*b*^	Fungi			
			Saccharomyces cerevisiae	NA	Fungi			
MOCK2	ZymoBIOMICS Gut Microbiome Standard	D6331	Akkermansia muciniphila		0.97	SRR13295152, SRR13295153		This study
			Bacteroides fragilis		9.94			
			Bifidobacterium adolescentis		8.78			
			Clostridioides difficile		2.62			
			Clostridium perfringens		0.0002			
			Enterococcus faecalis		0.0009			
			Escherichia coli	Escherichia flexneri	12.12			
			Faecalibacterium prausnitzii		17.63			
			Fusobacterium nucleatum	Fusobacterium animalis	7.49			
			Lactobacillus fermentum		9.63			
			Prevotella corporis		4.98			
			Roseburia hominis		9.89			
			Salmonella enterica		0.009			
			Veillonella rogosae		15.87			
			Methanobrevibacter smithii		Archaea			
			Candida albicans	NA	Fungi			
			Saccharomyces cerevisiae	NA	Fungi			

a Further details on the mock communities are available at https://www.zymoresearch.com/collections/zymobiomics-microbial-community-standards/products/zymobiomics-microbial-community-standard for D6300 and https://www.zymoresearch.com/collections/zymobiomics-microbial-community-standards/products/zymobiomics-gut-microbiome-standard for D6331.

b NA, Not applicable.

**FIG 4:**
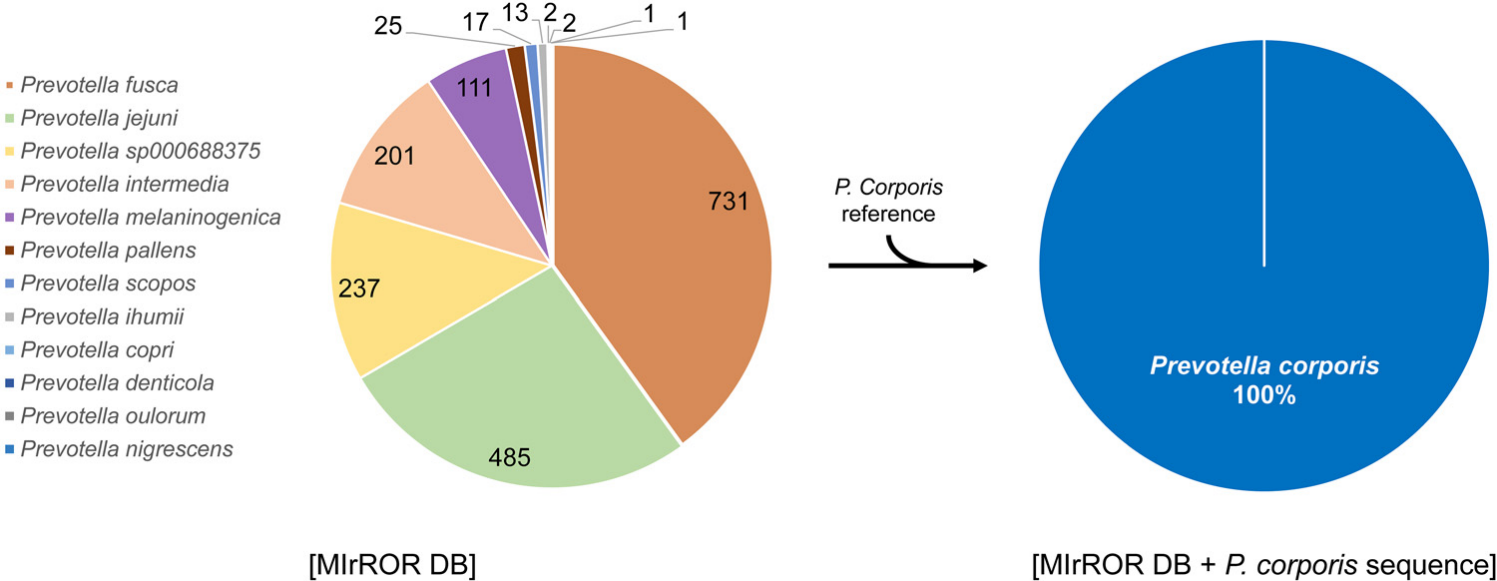
Abundance profile of reads classified as Prevotella at the genus level in MOCK2_1 community. (Left) Using the MIrROR database only. (Right) Reference 16S-ITS-23S rRNA operon sequences of P. corporis are added to the MIrROR database. The sequence identifiers were used to track changes in read classification caused by the addition of the reference.

**FIG 5:**
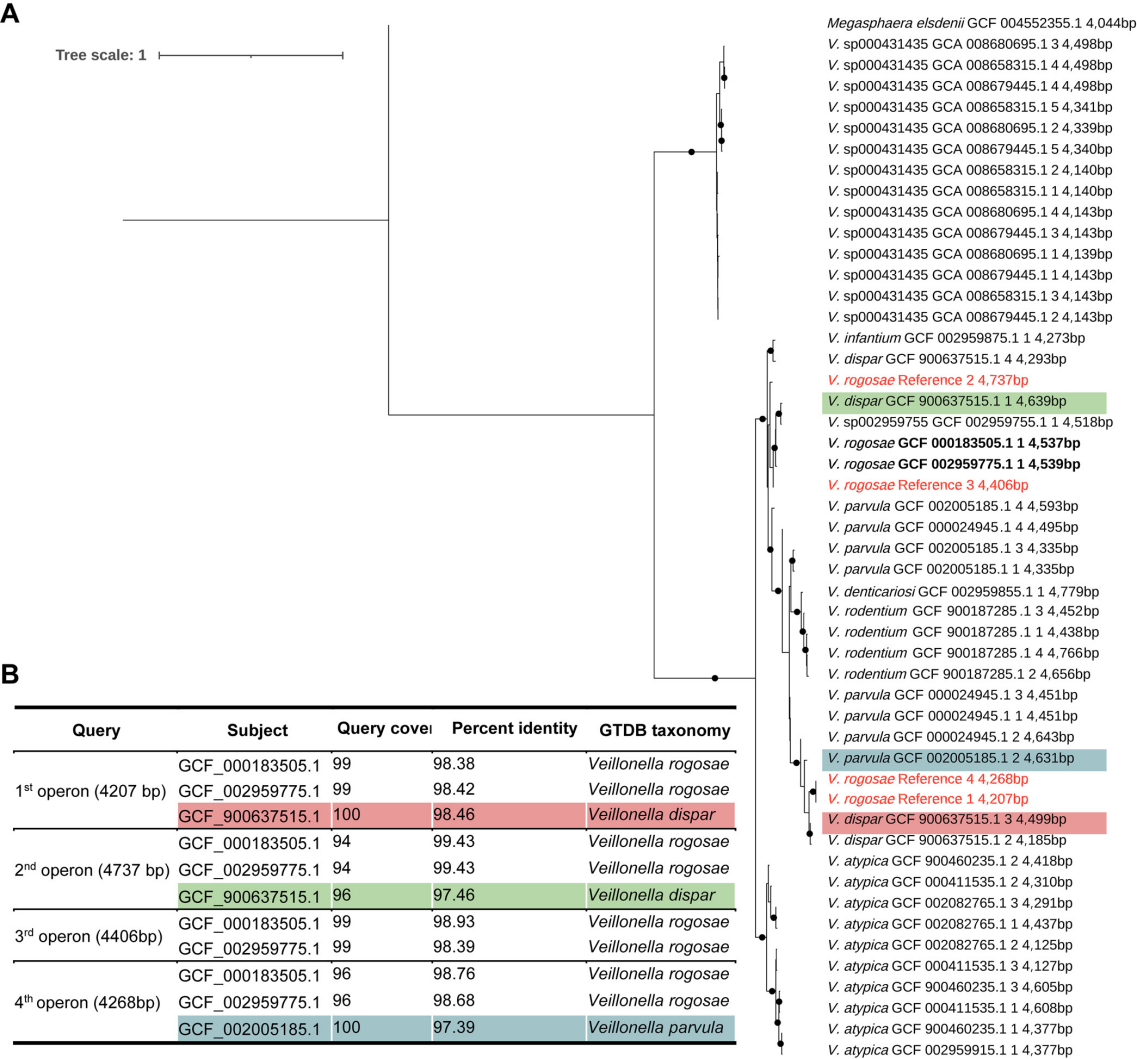
16S-ITS-23S rRNA operon relationship between V. rogosae (MOCK) and members of Veillonella genus from MIrROR. (A) Maximum likelihood phylogenetic tree for Veillonella 16S-ITS-23S rRNA operon sequences. Leaves for V. rogosae are indicated in bold, and the font color of the reference sequences is red. Labels for each leaf include GTDB species, NCBI accession number, the 16S-ITS-23S rRNA operon sequence count number within the genome, and sequence length. The bootstrap support value was set to 1,000, and nodes above 0.95 are depicted by circles. Megasphaera elsdenii was included as the outgroup. The tree was visualized with iTOL (72). (B) BLAST results for 16S-ITS-23S operon sequences of V. rogosae (reference) on the MIrROR database. The shaded subjects are the same as the shaded leaves in panel A.

In MOCK1, all species over 1% were correctly classified. In MOCK2, 15 species were detected to be more than 1%, of which six species were unexpected and were within the Prevotella or Veilonella genus (Table 3). Among the expected species in MOCK2, 9 out of 10 were correctly detected, excluding four species contained below 1% according to the theoretical composition. In the case of Prevotella corporis undetected species, there were only three genomes composed of many contigs in NCBI GenBank. Therefore, the 16S rRNA and 23S rRNA genes were not present together in one contig (Table 4), failing to extract the 16S-ITS-23S rRNA operon sequence. Therefore, reads were classified as closely related references such as Prevotella fusca (Fig. 4). The reason for the misclassified case, Veilonella, was that some of 16S-ITS-23S rRNA operon sequences of Veilonella rogosae from MOCK2 were more similar to those of other species than the reference sequence of V. rogosae in MIrROR (Fig. 5).

**TABLE 3:** False-positive species with relative abundance (RA) greater than 1%

Mock community	Related species’ theoretical abundance		MIrROR		16_mar		rrn_DBv2	
	Species	RA (%)	Species	RA (%)	Species	RA (%)	Species	RA (%)
MOCK1	L. fermentum	18.4					L. helveticus	2.6
							L. ginsenosidimutans	1.1
	None						Salinicoccus halodurans	3.3
							Shigella boydii	3.2
							Streptococcus pneumoniae	1.7
							Staphylococcus capitis	1.6
MOCK2	V. rogosae	15.9	V. dispar	6.2	V. parvula	6.9	Veillonella sp.	1.9
			V. parvula	3	Veillonella sp. oral clone AA050	5.1	V. atypica	1.8
	P. corporis	5	P. fusca	4.1	P. fusca	7.8	P. fusca	1.8
			P. jejuni	2.8	P. melaninogenica	1.5	P. intermedia	1.3
			Prevotella sp. 000688375	1.3			P. melaninogenica	1.3
			P. intermedia	1.1				
	F. nucleatum	15.9			Fusobacterium sp.	1.1		
	C. difficile	2.6			C. difficile shuttle vector pDSW1728	1		
	B. fragilis	10					B. ovatus	8.3
							B. uniformis	7.8
							B. fragilis	3.1
	None				Subdoligranulum sp. DJF_VR33k2	7.1		
					Ruminococcaceae bacterium D16	5.1		
							Propionibacterium propionicum	4.9
							Unclassified bacterium	2.9
							Streptococcus pneumoniae	2.8
							Bacillus thuringiensis	1.9
							Vibrio parahaemolyticus	1.8
							Rhodococcus erythropolis	1.5
							Klebsiella pneumoniae	1.3
							Geobacillus sp.	1
							Burkholderia cenocepacia	1

**TABLE 4:** Profile of rRNA genes for P. corporis genome

NCBI accession no.	Contig	rRNA gene	Position (strand)
GCF_000430525.1	NZ_AUME01000079.1	5S rRNA	3,028 to 3,113 (−)
	NZ_AUME01000091.1	23S rRNA	1 to 1,188 (−)
GCF_000613365.1	NZ_BAIT01000093.1	5S rRNA	49 to 157 (−)
	NZ_BAIT01000093.1	23S rRNA	342 to 3,234 (−)
	NZ_BAIT01000116.1	16S rRNA	2 to 1,250 (−)
GCF_001546595.1	NZ_KQ957193.1	23S rRNA	2 to 1,476 (−)
	NZ_KQ957224.1	16S rRNA	41 to 1,182 (−)
	NZ_KQ957299.1	16S rRNA	204 to 618 (+)

**FIG 6:**
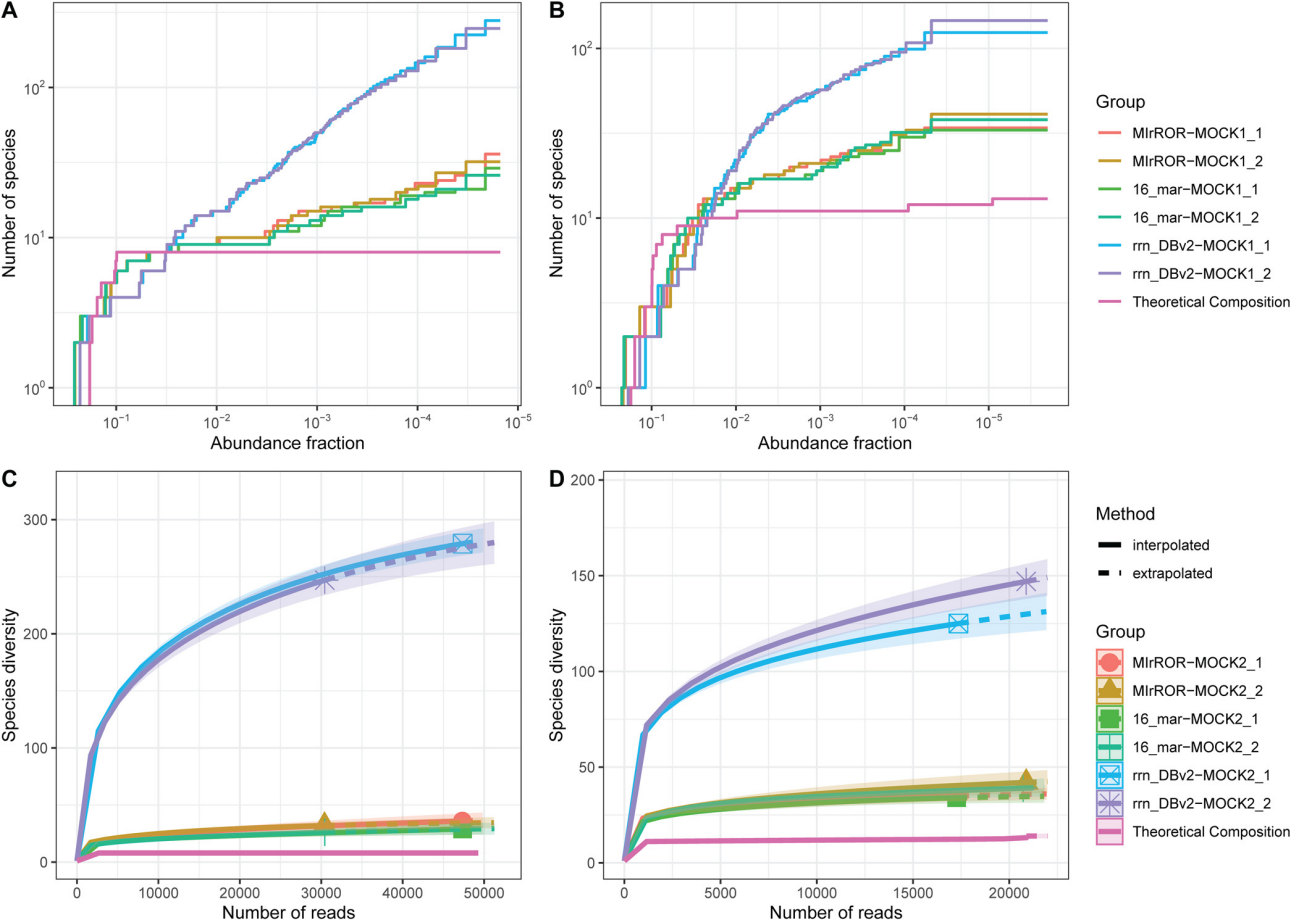
Performance comparison of community analysis for three databases. (A, B) Minimum abundance threshold for the number of classified species on the MOCK1 (A) and MOCK2 (B) communities. The theoretical fraction curve is depicted as a solid pink line. Note that the *x* axis is in reverse 10 log scale, and the *y* axis is in 10 log scale. (C, D) Rarefaction curves for the MOCK1 (C) and MOCK2 (D) communities. The shaded areas represent 95% confidence intervals.

We evaluated the accuracy of the above results compared to two databases: rrn_DBv2 and 16_mar. False-positive (FP) species continued to increase below 1% abundance in every database, regardless of community (Fig. 6A and B). MIrROR and 16_mar showed similar appearances, while rrn_DBv2 had more than 100 FP species. It was also confirmed in the rarefaction curve, and MIrROR and 16_mar converged with slightly higher species richness than MOCK communities (Fig. 6C and D).

**FIG 7:**
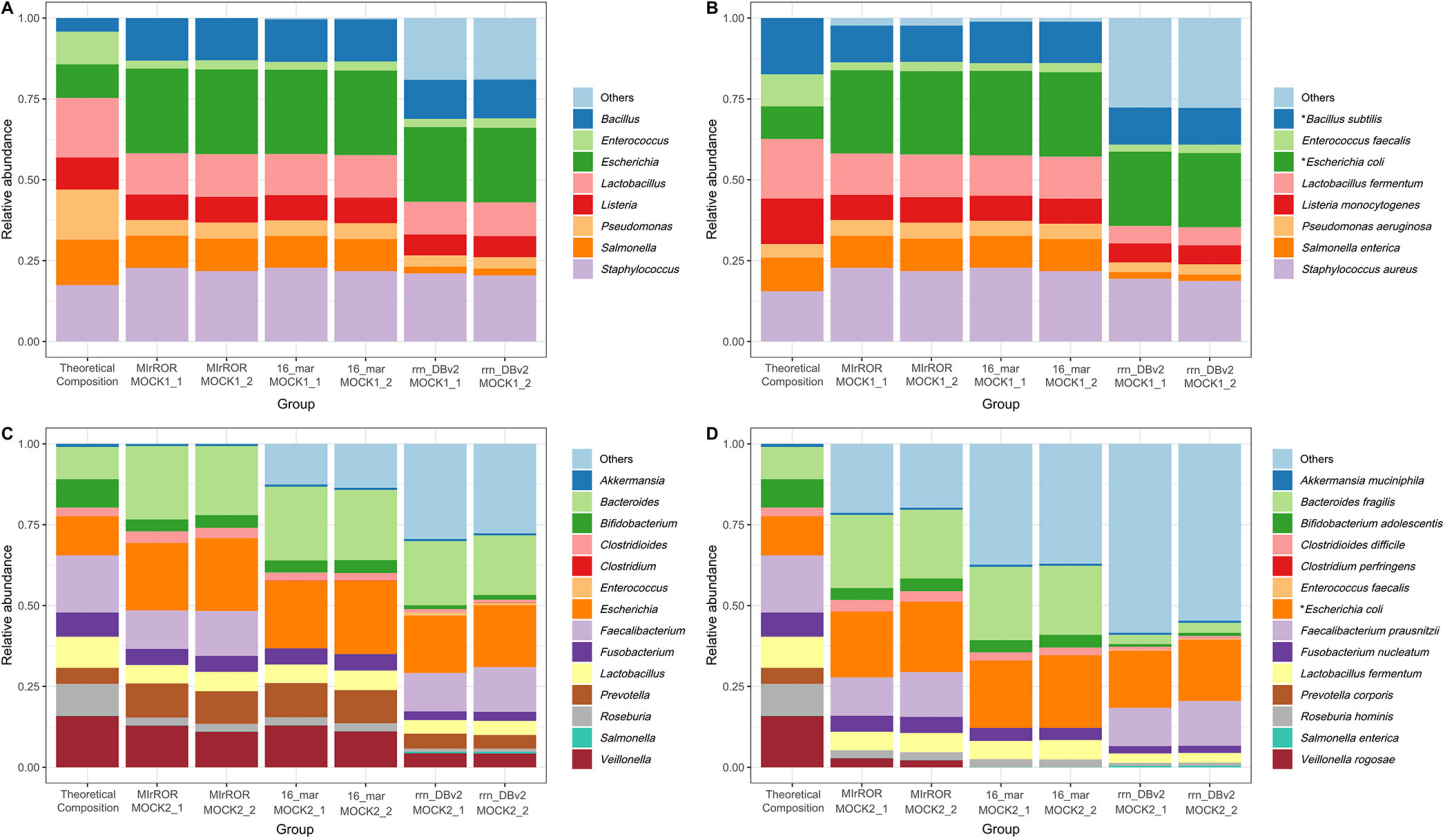
Taxonomic profiling results for MOCK community analysis with three databases. Relative abundances for the MOCK1 (A, B) and MOCK2 communities (C, D). Panels A and C and panels B and D are represented at the genus level and the species level, respectively. False-positive taxa belonged to “Others.” The asterisks in panels B and D indicate that MIrROR was used with taxonomic reassignment information; the relative abundance of E. coli and B. subtilis for MIrROR is expressed as the relative abundance of E. flexneri and B. marinus, respectively.

When using MIrROR as the reference database for investigating the MOCK1 community, only 4 and 1 reads (<0.01%) out of 47,327 (MOCK1_1) and 30,402 (MOCK1_2) were classified as unexpected taxa at the genus level, respectively. About 2.3% of the reads were misclassified at the species level. For 16_mar, about 0.4 and 1.1% of reads were misclassified at the genus and the species level, respectively. In the case of rrn_DBv2, there were no unclassified species among the MOCK1 species, but more than 20% of reads were classified as FP species (Fig. 7A and B; Table 3).

For MOCK2 community, MIrROR database had the lowest FP rate among the three databases. Despite the more complex community structure than MOCK1, only two reads were misclassified at the genus level in both MOCK2_1 and MOCK2_2 samples. However, the proportion of reads classified as “Others” was close to 20% at the species level for several reasons, including the absence of sequence of P. corporis. 16_mar did not have any sequence from the Faecalibacterium genus, so 14% of all classified reads failed to assign correctly even at the genus level. This value dropped even further to 37% of the total at the species level. When rrn_DBv2 was used, only 43% of reads were classified as the expected MOCK2 species (Fig. 7C and D; Table 3).

We performed taxonomic profiling analysis with the full-length 16S rRNA database instead of the 16S-ITS-23S rRNA operon database. 16S rRNA sequences extracted from 16S-ITS-23S rRNA operon reads were used in the analysis against the 16S rRNA database. When using the SILVA database with minimap2, they were assigned as an expected genus more than 97% of total reads but not more than 60% at the species level. Correctly assigned rates from the Greengenes database were inferior to those from the SILVA database at both the genus and species levels (Fig. S1), implying that it is difficult to expect species-level analysis when mapping the full-length 16S rRNA sequence with high error rates against the existing 16S rRNA databases (30).

We additionally compared the taxonomic profiling results for all databases using Kraken2, an alignment-free method. Taxonomy was assigned up to the genus level in the prebuilt SILVA database provided by Kraken2. Therefore, species-level analysis was not performed for the SILVA database. MIrROR and 16_mar had the lowest FP rates in genus and species level analyses of the MOCK1 sample, respectively (Fig. S2A and B). In MIrROR, about 10% of reads were misclassified as Bacillus velezensis rather than B. marinus. For MOCK2 samples, MIrROR showed the best performance among the databases (Fig. S2C and D). The FP rates of the MIrROR analysis tool and Kraken2 against the MIrROR database were as follows: MIrROR analysis tool versus Kraken2, 2.35% versus 17.25% (MOCK1_1), 2.31% versus 17.21% (MOCK1_2), 21.29% versus 20.89% (MOCK2_1), and 19.7% versus 21.96% (MOCK2_2). Despite using the same database, the number of observed taxa increased dramatically when using Kraken2 as follows: MIrROR analysis tool versus Kraken2, 37 versus 93 (MOCK1_1), 33 versus 82 (MOCK1_2), 35 versus 169 (MOCK2_1), and 42 versus 227 (MOCK2_2). Table S1 shows the time and the peak resident set size required to analyze the MOCK community using each tool and database.

### Assessing taxonomic analysis for human body sites.

A bacterial community in the real world is much more complex than the above mock community. However, it is inappropriate to use samples of unknown composition to evaluate databases or tools. Therefore, we generated *in silico* simulating data sets that mimic samples of human body sites where the majority of commensal bacteria are found. We constructed data sets through the genomes of 27, 18, 19, and 20 species of important (or major) bacteria found in the gut, skin, vagina, and oral cavity, respectively (Table S2). Among the three databases, MIrROR had the highest rate of correctly assigned, the highest Bray-Curtis similarity, and the lowest L2 distance (Table 5; Fig. S3). As in mock community analysis, more FP species than MIrROR arose in rrn_DBv2 and 16_mar for the following two reasons: (i) assignment to a closely related species because there is no corresponding species in the database and (ii) assignment to a closely related species due to error rates of insufficient curation.

**TABLE 5:** Benchmarking results on taxonomic profiling of four simulated data sets

Data set	Gut (2,700 reads)				Skin (1,800 reads)				Vagina (1,900 reads)				Oral cavity (2,000 reads)			
	Correctly assigned	Misassigned	Not assigned	L2 distance	Correctly assigned	Misassigned	Not assigned	L2 distance	Correctly assigned	Misassigned	Not assigned	L2 distance	Correctly assigned	Misassigned	Not assigned	L2 distance
MIrROR	2,502 (92.7%)	100 (3.7%)	98 (3.6%)	5.34	1,654 (91.9%)	103 (5.7%)	43 (2.4%)	7.89	1,738 (91.5%)	112 (5.9%)	50 (2.6%)	6.59	1,850 (92.5%)	96 (4.8%)	54 (2.7%)	7.01
16_mar	1,443 (53.4%)	1,164 (43.1%)	95 (3.5%)	18.33	1,445 (80.3%)	263 (14.6%)	92 (5.1%)	14.93	1,361 (71.6%)	489 (25.7%)	50 (2.6%)	16.58	1,366 (68.3%)	529 (26.5%)	105 (5.3%)	17.76
rrn_DBv2	909 (33.7%)	1,694 (62.7%)	97 (3.6%)	21.01	589 (32.7%)	1,119 (62.2%)	92 (5.1%)	26.16	536 (28.2%)	1,316 (69.3%)	48 (2.5%)	26.53	920 (46%)	974 (48.7%)	106 (5.3%)	22.52

## DISCUSSION

The 16S rRNA gene is a common target for Sanger sequencing when isolating and identifying bacteria. Numerous 16S rRNA nucleotide sequences have been registered in public repositories, whereas the 16S-ITS-23S rRNA operon sequences have not. Therefore, we extracted the 16S-ITS-23S rRNA sequences from the assembled genomes, including metagenome-assembled genomes, which have recently given us new insights (31, 32). However, we were concerned about their low quality (33, 34), so we performed several data curation and taxonomic reassignment. Genomes with any incorrect sequences were removed from the data set.

MIrROR included all 16S-ITS-23S operon sequences within a species if they differed even slightly. Therefore, the number of sequences in MIrROR may be exaggerated compared to other databases that have removed redundancies. However, we expect that sequences reflecting strains will be required soon. Analysis with strain-level resolution will be possible if the base quality of long-read sequencing is improved or unique molecular identifiers (UMIs) are used (20). Therefore, MIrROR did not remove redundancies by selecting the representative sequence of the cluster even if they were as similar as having 99% or higher sequence identity within the same species.

MIrROR, the highly curated database, not only has more sequences and taxonomic labels than other databases but also shows higher accuracy on community analysis. While two FP species unrelated to the MOCK community were found in 16_mar and 13 were found in rrn_DBv2, no irrelevant FPs were found in MIrROR, demonstrating that MIrROR has sufficient discrimination between species. Also, there was no case where any species was missing in the database, such as Faecalibacterium prausnitzii in 16_mar. It is unlikely that specific taxa were missing because we covered all available genomes from NCBI GenBank.

P. corporis was not covered in the MIrROR database. Due to absence of the genome, FP species corresponding to Prevotella were found. Of course, FP could be controlled by adding a reference sequence, but it is difficult to add a specific taxon’s sequence for every environment. It is possible to adjust the detection threshold to be more stringent. However, we could not simulate all cases, so we had no choice but to choose the robust option, which removes only obvious FPs. Kraken2 showed more FP species than the MIrROR tool regardless of whether the database contained the expected species. These results indicate that handling error-prone long reads with k-mer-based approaches that are sensitive to sequencing error is hard to control misclassification and is inappropriate for complex communities. Recently, several algorithms for long-read sequencing technologies, such as NanoCLUST (35), that cluster and classify reads based on Uniform Manifold Approximation and Projection (UMAP) and Hierarchical Density-Based Spatial Clustering of Applications with Noise (HDBSCAN), and MeTaPONT (36), which apply read classification (Centrifuge [37]) and alignment control together, have been developed, so we believe such problem will be resolved in the near future.

Further development of MIrROR will be focused on expansion. We provided MIrROR in a form that allows direct analysis by the most widely used universal primer sets. Therefore, the current release does not contain sequences amplified by the recently reported universal primer sets covering bacteria and archaea (38, 39). We plan to provide a ready-to-analyze database for newly reported regions, including archaea. We also expect to update MIrROR annually to reflect the growing number of newly assembled genomes and taxonomic information.

### Conclusion.

With MIrROR, researchers can solve the problem of low resolution derived from using only part of the 16S rRNA gene and can efficiently construct a high-resolution metagenome analysis pipeline by combining it with an economical sequencing platform such as Flongle. We expect that the bacterial community results obtained using MIrROR will contribute to various studies such as the Human Microbiome Project.

### Limitations.

Metabarcoding is the most efficient analysis technology that investigates the biodiversity of a community from a single bulk sample. However, researchers should keep in mind that there are two sides of the coin in amplifying only the marker gene in investigating community: (i) PCR amplification can result in erroneous compositional results due to primer bias, but it is an advantage for low-biomass density samples, such as milk or ascites, which have high host DNA content (40, 41); and (ii) while researchers cannot explore strain-specific gene contents or other domains of life, they can cost-efficiently monitor the community of the target domain. Therefore, it is necessary to comprehensively consider how much research funding is available for sequencing, whether functional analysis should be performed, and what the community’s characteristics are.

Regarding the last consideration, it may not be appropriate for some communities to use the 16S-ITS-23S rRNA operon sequence region as a taxonomic marker. Recent studies have revealed that unlinked rRNA genes are more frequent than expected, and the frequency increases in an environment where slower-growing or symbiotic taxa (such as Helicobacter, Rickettsia, Wolbachia, Buchnera, etc.) mainly live (42, 43), suggesting that community diversity cannot be fully covered by the 16S-ITS-23S rRNA operon region in such environments. Because the presence of unlinked rRNA genes was generally conserved at the species level, it is recommended to review the predominant species of the community before designing 16S-ITS-23S rRNA operon studies.

## MATERIALS AND METHODS

### Curating 16S-ITS-23S rRNA operon sequences.

We downloaded all of the latest versions of bacterial genomes, including those that were not RefSeq, available from NCBI GenBank according to the guideline (https://www.ncbi.nlm.nih.gov/genome/doc/ftpfaq/) (44). After collecting bacterial genomes, we generated *in silico* amplicon product of the 16S-ITS-23S rRNA operon using EMBOSS-primersearch (version 6.6.0.0) (45) and universal primer set (16S-27F: 5′-AGRGTTYGATYHTGGCTCAG-3′ and 23S-2241R: 5′-ACCRCCCCAGTHRAACT-3′). In consideration of possible variants in the primer binding site, a maximum of two mismatches were allowed. Filtering and curation were performed according to the following criteria: (i) Product size was between 3,500 and 7,000 bp. (ii) Genomes containing ambiguous nucleotides other than A, T, G, and C in the 16S-ITS-23S rRNA operon sequence were removed. (iii) For genomes that had sequences that met the previous two criteria, GTDB-Tk (version 1.2.0) was performed to prevent taxonomy misassignment (46). If genomes had an insufficient percentage of amino acids for the GTDB-Tk process or were not assigned to the species level, we removed it from the data set. An alphabetic suffix of GTDB taxonomy was not considered. (iv) We removed sequence duplicates from the same species. (v) If the 16S-ITS-23S rRNA operon sequences were similar or identical between different species, we identified the wrong side. For this step, we clustered the sequences with cd-hit-est (version 4.8.1) at 99% identity (47). When two or more taxa existed in a cluster, contamination was checked by mapping the sequences with the minor taxon (derived from less than two genomes) to the database after step iv using BLAST (version 2.10.1) (48). When a minor taxon could not be distinguished, especially if it consisted of two species within the genus, we constructed a phylogenetic tree with all sequences within the genus using Clustal Omega (version 1.2.4) (49) and IQ-TREE (version 2.0.6) (50) with a default option to determine whether it was contaminated or not. After that, we performed BLAST on all-to-all to check again whether genomes of other species were hit within the top 100 hits for each genome. Sequences suspected of being contaminated were finally confirmed by webBLAST (51) against nucleotide collection (nr/nt). If a genome contained a contaminated sequence, the correct sequences in that genome were also removed. For gene copy normalization, the average rRNA operon copy number for each taxon was calculated in step iii.

### rRNA operon project.

To collect sequencing data of reported 16S-ITS-23S rRNA operon studies and construct taxonomic profiling results, the following search terms were entered into NCBI BioProject: “rRNA” [All Fields] AND “operon” [All Fields] OR “rrn” [All Fields] AND “operon” [All Fields] OR “rrn” [All Fields] OR “16S-23S” [All Fields] AND “rRNA” [All Fields] OR “16S-ITS-23S” [All Fields] AND “rRNA” [All Fields]. The metadata of each run were retrieved using esearch and efetch from Entrez Direct (version 13.9) (52). Publications related to each project were found by searching BioProject accession on PubMed. For 16S-ITS-23S rRNA operon analysis, we used a long-read mapper, Minimap2 (version 2.17) (53). Secondary alignment was not considered to reduce FP caused by high error rates. By default, only the number of residue matches (≥2,500 bp) and alignment block length (≥3,500 bp) were considered, as suggested by Cuscó et al. (18). The profile of each sample were drawn with KronaTools (version 2.71) (54) and Google Charts (https://developers.google.com/chart). MIrROR’s analysis tool is detailed in the Code Availability section.

### Web server.

MIrROR website was implemented in Apache, Django framework, SQLite, and Python. The web-based user interface is written in HTML5, CSS, and JavaScript. We also provided BLAST for calculating sequence similarity between query sequences and MIrROR database.

### Evaluation and comparison of databases.

Reported 16S-ITS-23S rRNA operon databases include rrn_DB (17), rrn_DBv2 (updated version of rrn_DB) (27), 16S-23S-rRNA encoding region database (16_mar) (28), ncbi_202006 database (DB) (39), and Athena database (55). Among those, publicly available databases rrn_DBv2 and 16_mar were used to evaluate the performance of MIrROR.

First, a sequence-level evaluation was performed using RESCRIPt (version 2021.4.0), a plugin from Quantitative Insights into Microbial Ecology 2 (QIIME2) (56, 57). To compare the sequences used for community analysis, we extracted the amplicon region targeted by 16S-27F and 23S-2241R primer set from all databases. We used the “evaluate-seqs,” “evaluate-taxonomy,” and “evaluate-fit-classifier” actions to evaluate the amount of information in the database and the accuracy as a naive Bayes-based classifier.

Second, we evaluated MIrROR’s performance at the community-analysis level using Nanopore metagenomic data. We used ZymoBIOMICS microbial community standard (catalog no. D6300; MOCK1) and gut microbiome standard (catalog no. D6331; MOCK2) as mock communities. Taxonomic reassignment was performed using the whole genome of each strain provided by the company.

For MOCK2, DNA was extracted as described previously with 0.5-g zirconia beads (0.1- and 0.5-mm diameter, Bio Spec Products Inc.) (58). We added beads to a sample tube and then vortexed it for 5 min at maximum speed on Vortex-Genie 2 mixer. The vortexed sample was centrifuged at 3,000 × *g* for 15 min, and the supernatant was transferred into a 2-mL tube containing 0.75 vol of isopropanol, vortexed, and loaded on the DNA-binding column (Bioneer). The purity and concentration of DNA were quantified using Synergy HTX multimode reader and Gen5 software (Biotek). To amplify the bacterial rRNA operon, the following primers that were modified and tailed with ONT barcode sequence were used: 16S-27F-GGTGCTG-ONT barcode sequence, TTAACCTAGRGTTYGATYHTGGCTCAG; and 23S-2241R-GGTGCTG-ONT barcode sequence, TTAACCTACCRCCCCAGTHRAACT (17, 18). For PCR, 50 ng of DNA template was amplified in 50 μL reaction volume using the BX-Tag polymerase (Bionics) in the following thermal condition: an initial denaturation of 2 min at 98°C, followed by 25 cycles at 10 s at 98°C, 30 s at 50°C, 150 s at 72°C, and a final step of 10 min at 72°C. PCR amplicon was confirmed by agarose gel electrophoresis and cleaned with SPRIselect beads (Beckman Coulter) with 0.45× ratio. Cleaned amplicon sample was quantified using a Quant-iT PicoGreen double-stranded DNA (dsDNA) assay kit (Invitrogen). The library was prepared using the ONT one-dimensional ligation sequencing kit (SQK-LSK109, Nanopore) in accordance with the manufacturer’s protocol. In brief, barcoded DNA amplicons were pooled in equal molar amounts (total of about 100 to 200 fmol) and repaired using the NEBNext FFPE DNA Repair Mix and NEBNext Ultra II End Repair/dA-Tailing Module (NEB) at 22°C for 30 min. End repaired sample was ligated with a Nanopore adapter using the NEBNext quick ligation module at room temperature (RT) for 10 min. After every enzyme reaction, the DNA samples were purified using AMPure XP beads (Beckman Coulter) according to the manufacturer’s instructions. The final library was loaded onto Flongle flow cell (FLO-FLG001, R9.4.1), and sequencing was performed for ∼30 h on a MinION MK1b and MinKNOW software (version 19.12.5).

Fast5 files generated from Flongle were base-called using Guppy (version 4.0.11). Demultiplexing and adapter trimming were performed with Porechop (version 0.2.4) (59). Trimming was also performed on MOCK1 sample. After the trimming, we used NanoFilt (version 2.7.1) to remove reads with sizes outside the range of 3,500 to 5,000 bp (60). For 16S-ITS-23S rRNA operon analysis, MIrROR’s analysis tool with default option was performed with only the reference database changed from MIrROR database to rrn_DBv2 and 16_mar for comparison.

For comparison with full-length 16S rRNA analysis, the 16S rRNA sequences were extracted from 16S-ITS-23S rRNA operon reads using metaxa2 (version 2.2) (61) and aligned against the SILVA 138.1 NR99 references (22) and the Greengenes 13_8 97% reference sequences (23) using the MIrROR tool with the following parameters: -m 800 -b 1,100.

For comparison between MIrROR tool and Kraken2 (version 2.1.2) (29), we restructured the 16S-ITS-23S rRNA operon databases so that they can be used in script “build_gg_taxonomy.pl.” Then, we built Kraken2 database using script “16S_gg_installation.sh” (https://github.com/DerrickWood/kraken2/tree/master/scripts/). Prebuilt Kraken2 databases (Kraken 2 16S Greengenes 13_5 DB and Kraken 2 16S Silva 138 DB) were downloaded for 16S rRNA analysis (ftp://ftp.ccb.jhu.edu/pub/data/kraken2_dbs/). Kraken2’s read classification was performed using default options for each database. GNU time (version 1.7) was used to calculate execution times and memory peaks. Each task was given 50 threads.

The rRNAs were predicted for P. corporis using barrnap (version 0.9) (https://github.com/tseemann/barrnap). The phylogenetic tree for Veillonella was generated using IQ-TREE (version 2.0.6) (50) with ultrafast bootstrap. Sequence identities for 16S-ITS-23S rRNA operon sequences were obtained by using BLAST (51). Rarefaction curve for species richness was obtained using the iNEXT R package (version 2.0.20) (62). Stacked bar plot was drawn with ggplot2 R package (version 3.3.5) (63).

Third, we evaluated whether MIrROR could be used to investigate gut, skin, vaginal, and oral bacterial community. For read simulating, the main species of each body site was selected (64–67). As in the construction of the MIrROR database, the 16S-ITS-23S rRNA operon sequences were extracted from each genome, and reads were simulated using NanoSim (version 3.0.0) (68). MOCK2_1 sample was used to build the error profile. A total of 100 reads by species were concatenated for each body site and then aligned against MIrROR, rrn_DBv2, and 16_mar database as in mock community analysis. We calculated the L2 distance to compare the expected relative abundance for data sets with estimated abundance from each analysis task. The L2 distance was calculated as shown below (69).

$$L_{2}\;\text{distance}=\;\sqrt{\overset{n}{\underset{i}{\sum }}{\left(\text{estimate}d_{i}-\text{expecte}d_{i}\right)}^{2}}$$

Principal coordinate analysis was conducted based on a Bray-Curtis dissimilarity matrix using the phyloseq R package (version 1.38.0) (70).

### Data availability.

The nanopore sequence data generated for this study are deposited in the NCBI Sequence Read Archive (SRA) and available through accession number BioProject PRJNA687502.

### Code availability.

MIrROR’s analysis tool, which is implemented in Python, is freely available at https://github.com/seoldh/MIrROR. It uses a long-read mapper, Minimap2 (53). Secondary alignment is not considered to reduce false positives caused by high error rates. By default, only alignment block length (≥3,500 bp) and the number of residue matches (≥2,500 bp) are considered. The profile of each sample and distribution of the relative abundance of taxa between groups are drawn with KronaTools (54) and matplotlib (71).
