# Supplementary material for: Microbial Identification Using rRNA Operon Region: Database and Tool for Metataxonomics with Long-Read Sequence
Source: Microbiol Spectr. 2022 Mar 30;10(2):e02017-21. doi: 10.1128/spectrum.02017-21 (PMC9045266; doi:10.1128/spectrum.02017-21)
Supplement: SUPPLEMENTAL FILE 2 — Supplemental material. Download SPECTRUM02017-21_Supp_1_seq9.pdf, PDF file, 0.5 MB [file spectrum02017-21_supp_1_seq9.pdf]

Supplementary Materials for

Microbial Identification using rRNA Operon Region: database and tool for meta-taxonomics with long-read sequence

Donghyeok Seol, Jin Soo Lim, Samsun Sung, Young Ho Lee, Misun Jeong, Seoae Cho, Woori Kwak, and Heebal Kim*

*Corresponding author: Heebal Kim (heebal@snu.ac.kr)

SUPPLEMENTAL FIGURES

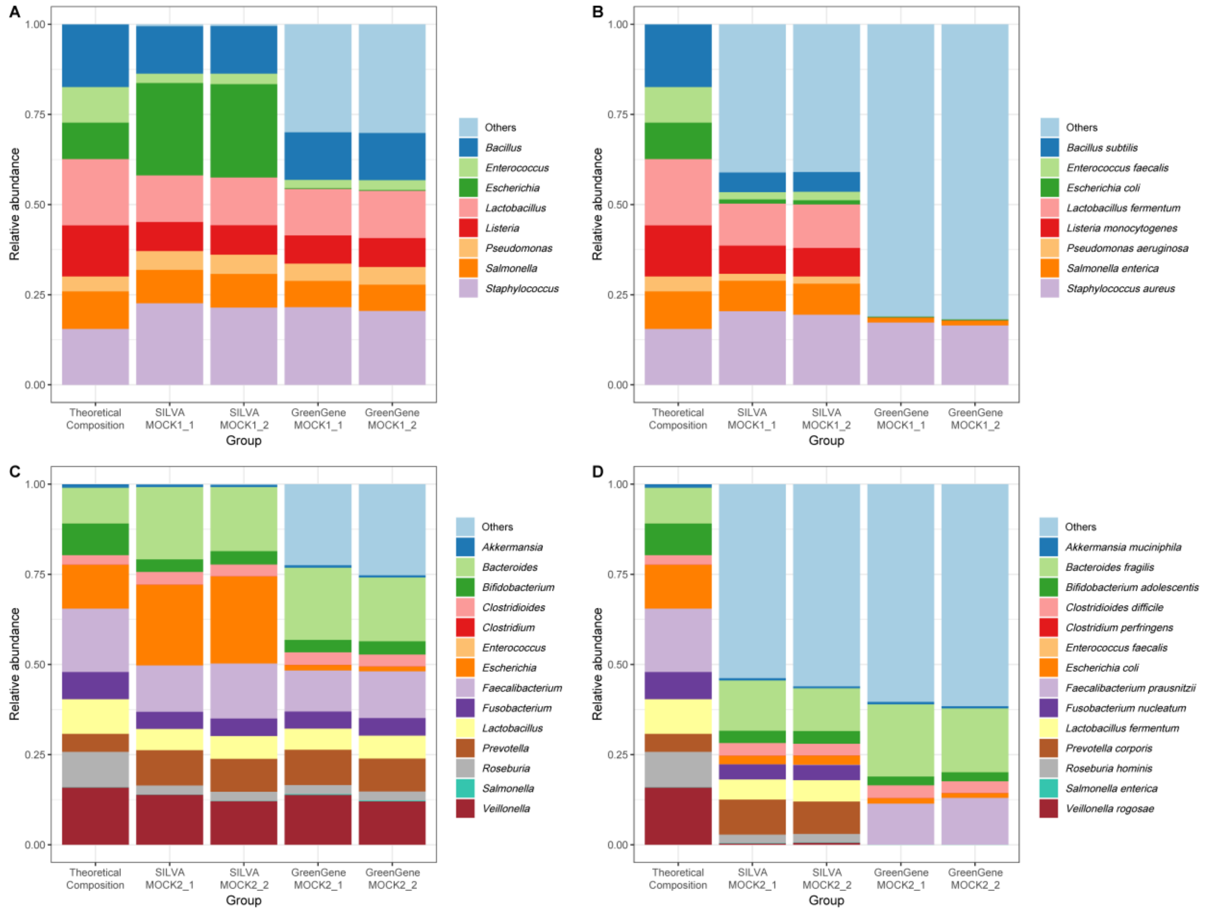

Supplementary Figure 1. Taxonomic profiling result with 16S rRNA database.

Relative abundance for (A and B) MOCK1 and (C and D) MOCK2 community. (A and C) and (B and D) are represented at the genus level and the species level, respectively. False positive taxa belonged to ‘Others’.

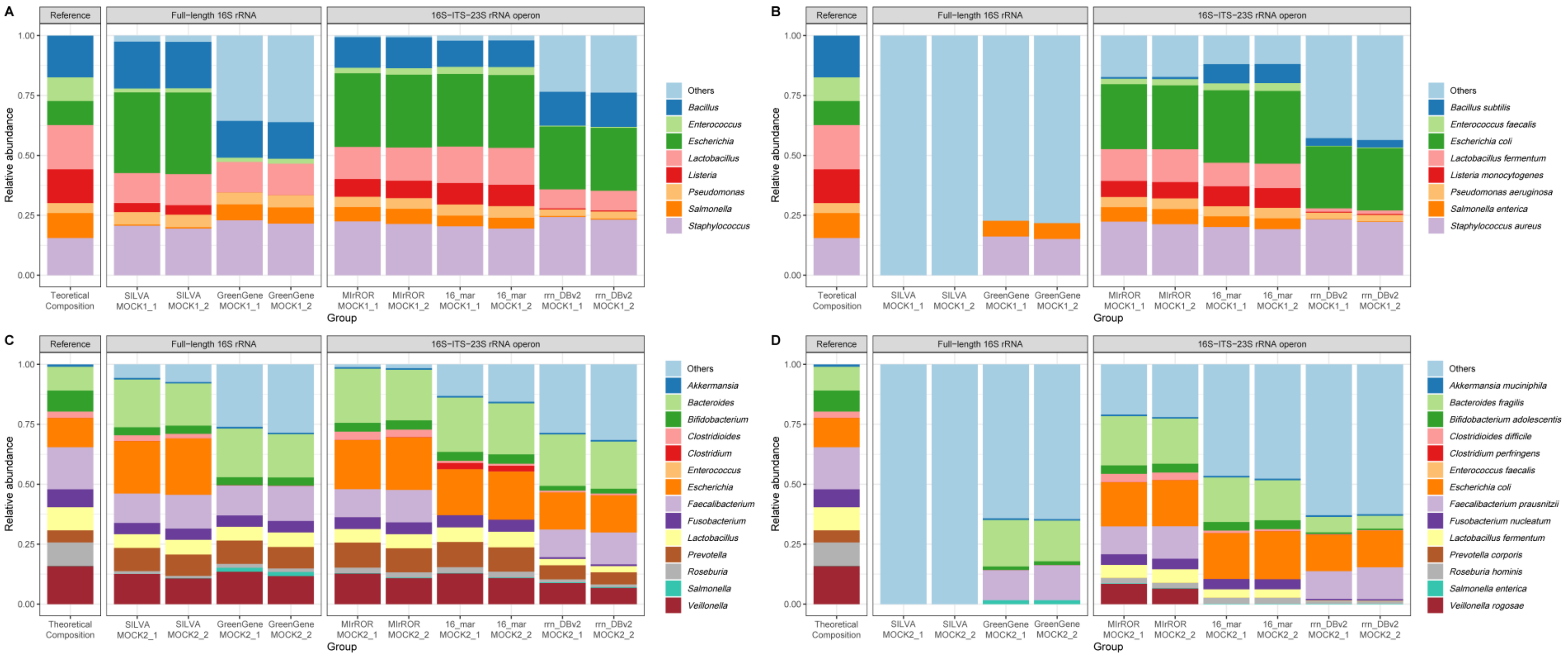

Supplementary Figure 2. Taxonomic profiling result with Kraken2.

Relative abundance for (A and B) MOCK1 and (C and D) MOCK2 community. (A and C) and (B and D) are represented at the genus level and the species level, respectively. False positive taxa belonged to ‘Others’.

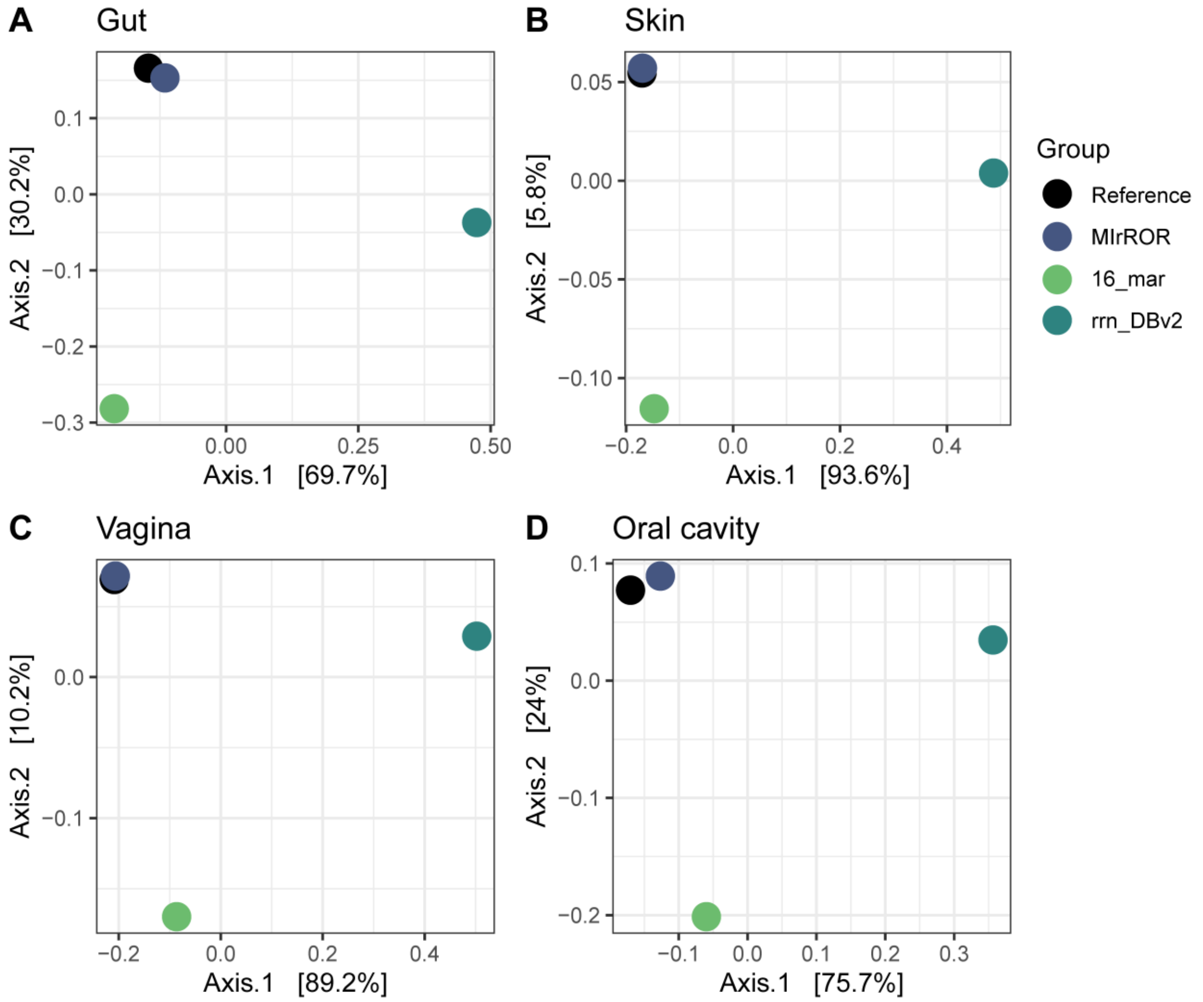

Supplementary Figure 3. Principal coordinate analysis (PCoA) based on Bray-Curtis distances between the three 16S-ITS-23S rRNA operon databases.

(A) Gut, (B) Skin, (C) Vagina, and (D) Oral cavity.

SUPPLEMENTAL TABLE

Supplementary Table 1. Analysis times and memory peaks by tools and databases.

Database	MOCK1_1		MOCK1_2		MOCK2_1		MOCK2_2	
	MIrROR tool	Kraken2	MIrROR tool	Kraken2	MIrROR tool	Kraken2	MIrROR tool	Kraken2
SILVA	3110%/35.94/27.9	-	4054%/17.18/19	-	1856%/16.33/9.2	-	3770%/08.03/9.3	-
GreenGene	3534%/15.02/12.5	735%/01.05/0.7	4084%/07.65/8.4	1202%/00.45/0.5	3993%/03.75/4.4	1215%/00.33/0.4	3916%/03.92/4.4	1200%/00.36/0.4
MIrROR	4828%/7:42.23/175.7	2427%/00.84/1.6	4773%/5:03.04/120.4	1874%/00.75/1.3	4059%/1:27.92/55.1	1199%/00.79/1	3997%/1:33.48/56.3	1570%/00.66/1.2
16_mar	4728%/2:45.93/55.2	2119%/00.79/1.3	4671%/1:48.41/37.5	1697%/00.68/1.1	3917%/34.04/16.8	1264%/00.61/0.9	3968%/36.09/17.8	1544%/00.55/1
rrn_DBv2	4790%/2:44.65/54.5	2027%/00.77/1.3	4675%/1:47.51/37	1715%/00.64/1.1	4035%/31.89/16.8	1321%/00.54/0.8	3882%/35.89/17.8	1702%/00.46/0.8

Data are presented as ‘Percent of CPU this job got’ / ‘Elapsed time (m:ss)’ / ‘Maximum resident set size (GB)’
